# An Evaluation of the Fracture Properties of Asphalt Concrete Mixes Using the Semi-Circular Bending Method and Digital Image Correlation

**DOI:** 10.3390/ma18050967

**Published:** 2025-02-21

**Authors:** Piotr Zieliński, Marek Klimczak, Marcin Tekieli, Mateusz Strzępek

**Affiliations:** Faculty of Civil Engineering, Cracow University of Technology, 31-155 Cracow, Poland; marek.klimczak@pk.edu.pl (M.K.); marcin.tekieli@pk.edu.pl (M.T.); mateusz.strzepek@pk.edu.pl (M.S.)

**Keywords:** semi-circular bending (SCB), reclaimed asphalt shingles (RASs), asphalt concrete (AC), digital image correlation (DIC), flexibility index (FI), cracking resistance index (CRI)

## Abstract

The semi-circular bending method (SCB) is a useful test for evaluating the cracking resistance of asphalt mixtures with added reclaimed asphalt shingles. A mixture of the asphalt concrete AC 16 with 50/70 paving bitumen was used for the binder course test as a reference mix. The purpose of the paper is to evaluate two aging conditions (short-term and long-term) of the above-mentioned asphalt mixtures in relation to their fracture properties. Laboratory experiments are enhanced with the application of image processing techniques (digital image correlation and image segmentation) that account for the asphalt mixture heterogeneity. Consequently, they can provide a more detailed description of the specimen performance. Statistical analyses of the laboratory results indicate that the best sensitivity in terms of differentiating the tested mixtures, especially taking into account the aging conditions of the mixtures, was observed for the post-peak parameters such as the flexibility index (FI), toughness index (TI), and, above all, cracking resistance index (CRI), for which the average coefficient of the result variability is approximately 10%, while for the FI and TI parameters it is approximately 30%. Digital image correlation analyses provided a confirmative illustration of the aforementioned observation.

## 1. Introduction

Efficient management of resources during road construction can be achieved by, among other strategies, increasing the use of recycled materials [1,2,3]. Testing on the use of reclaimed asphalt shingles (RASs) as an asphalt concrete (AC) component has been conducted for almost forty years [4]. The application of RASs in AC mixes generally shows an improved resistance of asphalt mixtures at high-temperature conditions by reducing the depth of ruts in the pavement [5,6,7,8]. Simultaneously, mixtures with RAS addition may be more sensitive to cracking, both at low pavement operating temperatures [3,6] and due to fatigue [3,5]. As was stated by Ozer et al. [9], mixtures with an increased content of the RAS binder (more than 40% of the total binder) are more susceptible to fatigue cracking as well as low-temperature cracking, compared to mixtures with a typical RAS binder content (about 20%).

Referring to the origin of RAS, there are two types of materials: manufactured waste asphalt shingles (MWASs), which contain bitumen that has not been subjected to operational aging, and tear-off asphalt shingles (TOASs), which are material stripped from old roofing. According to the work of Zhou et al. [6], asphalt mixtures containing added TOASs are more susceptible to low-temperature cracking than those with the MWASs added. This is because the binders contained in TOASs are highly oxidized due to long-term exposure to weather conditions during use as a roof covering [10,11]. Additionally, mixtures with TOASs, compared to those with added MWASs, are also more susceptible to damage caused by water action [12]. The solution to fix the above-mentioned problems is the application of the new softer bitumen and reducing the RAS ratio, especially during TOAS application [6]. Another quite popular application in the RAS mixtures is the use of refreshing additives, but not all of them are effective [13]. The positive results for mixtures with RASs added using the wet method in terms of fatigue as well as low-temperature cracking were confirmed in a publication made by Alvergue et al. [14] and Elseifi et al. [15]. Additionally, test results [16,17] indicate that RAS mixtures are generally characterized by a lower temperature sensitivity in terms of the complex stiffness modulus than the reference mixture.

In general, in the case of mixtures with RASs, low-temperature cracking and fatigue resistance should be tested. One of the test methods useful for testing the intermediate service temperature cracking performance of asphalt pavements is semi-circular bending (SCB) [15,18]. The results achieved by Ozer et al. [19] confirmed the applicability of the SCB test results for the evaluation of the AC fatigue properties. Of the many parameters of this test describing fatigue properties of asphalt mixtures, it is not recommended to use the peak load [20]. The post-peak slope parameters better differentiate mixtures with different compositions and aging conditions [21], as is also the case in assessing the mixtures with portions of recycled pavement material [22]. A popular parameter describing fracture resistance and sensitivity to the influence of various factors in mixtures better than just fracture energy is the flexibility index (FI) [23]. As shown by the results of [24], FI significantly differentiates mixtures, due to the effective volume of the binder, the low-temperature grade of the virgin binder, the ratio of the recycled binder, the application of polymers for bitumen modification, and aging conditions. A practical advantage of using the flexibility index is that it has a good correlation with fatigue cracking results [25,26]. As was concluded in the work performed by Zhang et al. [27], the FI value is a good parameter in identifying asphalt mixture cracking performance. Chen [28] stated that the application of recycled materials containing bitumen in a new asphalt mixture significantly increases the maximum load as well as decreases the FI value during the SCB test, especially for asphalt mixtures aged in the long-term [28]. As was stated by Zieliński [29], the flexibility index as well as the cracking resistance index (CRI) are the best parameters to describe the impact of asphalt mixture aging conditions in terms of fracture toughness. The CRI parameter, compared to the flexibility index, has the following advantages: simpler calculations, smaller result scatter, and better characterization in the case of brittle mixtures [30]. Ren et al. [31] also confirmed that the CRI provides better repeatability of the results than the FI parameter. As was stated in the work of Majidifard et al. [32], the newly introduced parameter, the “balanced cracking index” (BCI), had the best correlation with real road section data, which allows this parameter to be used to assess the cracking potential of the asphalt pavement layer. Moreover, it was shown that the BCI is characterized by a low dispersion of results, which makes it easier to demonstrate the significance of differences between the tested mixtures in statistical tests.

The selection of appropriate conditions for performing the SCB test in terms of temperature and load rate should consider the climatic regional conditions [33]. The optimal conditions for the SCB test should minimize the scatter of results; e.g., in the work conducted by Nsemgiyumva, Kim and You [34], they are defined as follows: temperature of test—15–40 °C, load rate—1 mm/min to 5 mm/min, number of samples—5 to 6, depth of notch—5 mm to 25 mm, and sample thickness—40 mm to 60 mm. Ozer et al. [35] proposed a minimum of six samples in the SCB test as the recommended value.

The results of laboratory and FEM simulation tests of SCB samples of asphalt concrete subjected to weather conditions (water and frost action) and aging procedures were presented by Szydłowski et al. [36]. The authors stated that enhancing laboratory test results with a limited number of tested samples via numerical simulations ensures data extension for more reliable statistical analyses and estimates of the reliability of the material designed.

The purpose of this paper is to evaluate two aging conditions (short-term and long-term) of the asphalt mixtures (with RAS addition and reference), in relation to their fracture properties, as calculated from the SCB test with DIC enhancement. Using statistical analyses, the most sensitive parameters of the SCB test were identified, which allowed for the differentiation of the tested mixtures.

## 2. Materials and Methods

**Figure 1:**
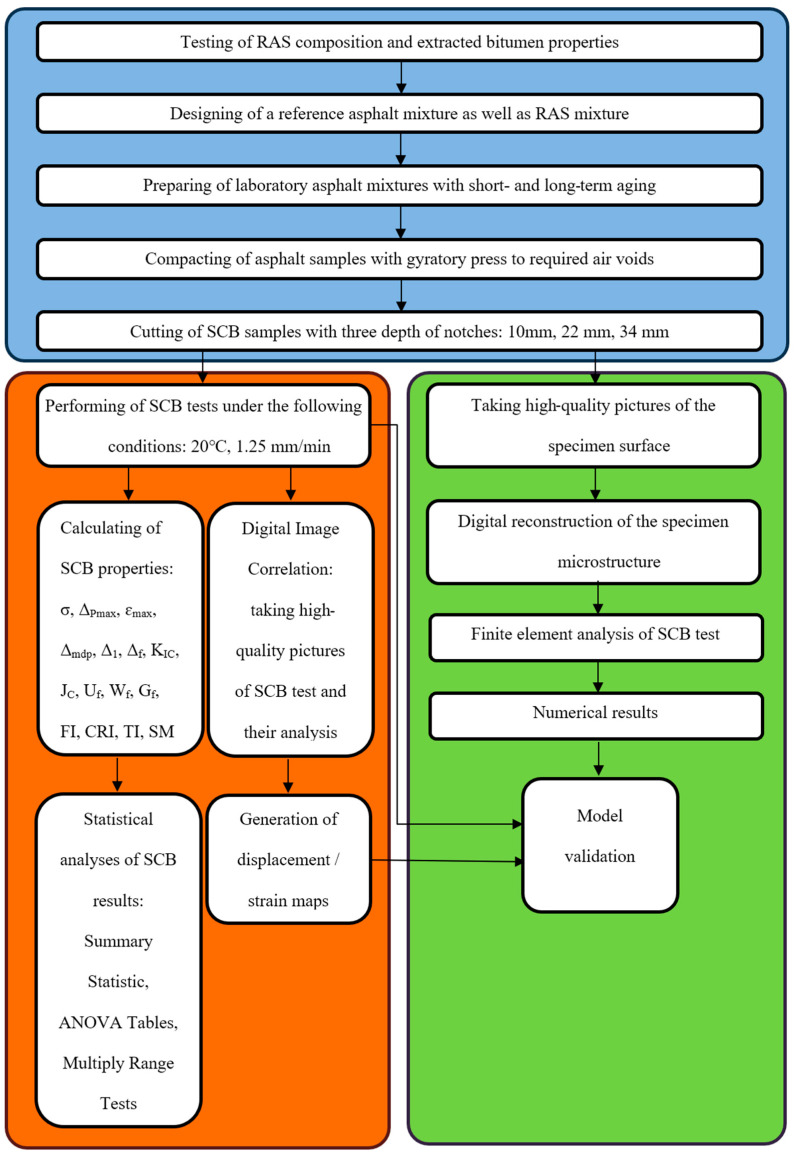
Flow chart for program of tests.

As a reference for testing, the mixture of the asphalt concrete AC 16 for the binder course was used. For comparison, a mixture with added manufactured asphalt shingles was prepared. For all tested mixtures, 50/70 bitumen was used as the fresh binder. The test program is shown in Figure 1. We marked three main phases of the test with different colors. The phase of the sample preparation is marked with blue. It comprises five steps that end with the preparation of SCB samples with three depths of notches. Subsequently, two branches can be distinguished. The classical SCB test (left outermost branch of the flowchart) is enhanced with a DIC analysis (neighboring branch). These two approaches (marked together with orange) aim to quantify the real SCB test results. The standalone SCB test provides macroscopic measures for the specimen. When enhanced with the DIC analysis, the microscopic phenomena can also be studied. These two approaches, marked with independent branches in the flow chart, are supplementary. The branch marked with green describes the virtual experiment performed after the digital reconstruction of the specimen microstructure. The finite element method is used for this purpose. In this paper, the initial results of the analysis of the linear elastic range are presented. In our future research, nonlinear models (e.g., [37]) will be used to describe the response of the specimen. It is important that all three analyses (classical SCB test, DIC analysis and finite element analysis, FEA) are not standalone approaches but complement each other. In particular, FEA requires model validation that is performed using the results of the two remaining approaches. In the flow chart, it is schematically illustrated with two corresponding links to the “Model validation” block. At this step, the adopted material models and their parameters can be validated based on the real experiment results.

The grain size composition of RASs after bitumen recovery, as well as extracted binder properties, are given in Table 1.

Both tested mixtures were designed according to the same grain size curve as well as the same total bitumen content. The ingredients of the above-mentioned mixtures are presented in Table 2, while the grain size of the mineral mixture and the extracted binder content are listed in Table 3.

**Figure 2:**
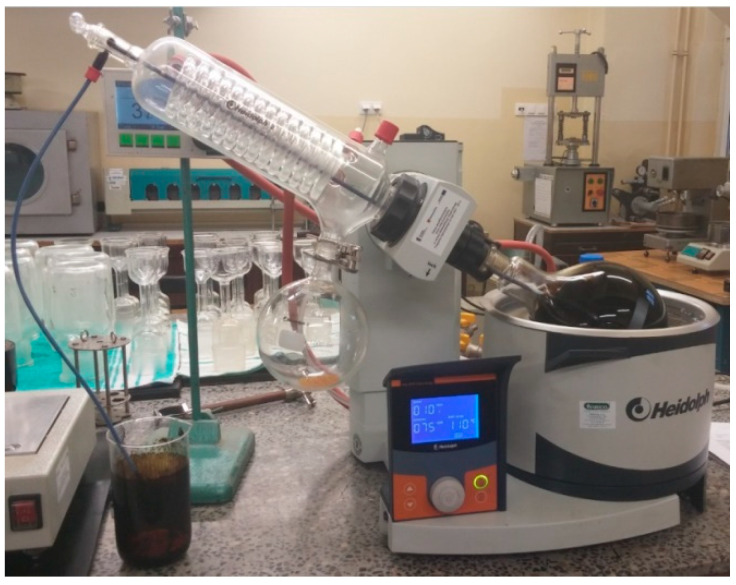
Rotary evaporator during distillation of solvent from bitumen–solvent mixture.

The preparation of asphalt mixtures in the laboratory consists of dosing the RAS, heated to 60 °C, into a hot aggregate at a temperature of approx. 180 °C, mixing and then adding a 50/70 bitumen binder at a temperature of approx. 150 °C, and finally mixing the mixture once again. After final mixing, samples were taken from each mixture to test the composition in an ultrasonic extractor and density in a pycnometer. The bitumen–solvent mixture obtained as a result of extraction was distilled in a rotary evaporator (Figure 2) in accordance with the EN 12697-3 standard [38], where, under the influence of increased temperature and reduced pressure, the solvent was condensed and pure asphalt was obtained from the tested mixtures. The bitumen was poured through a sieve to remove air bubbles and then samples were prepared in accordance with the required functional standards for testing: penetration, softening point and ductility with force measurement. The test results for the binder recovered from the mixtures in question, as well as the fresh bitumen, are presented in Table 4.

Before compacting the samples, the loose mixtures were subjected to short-term and long-term aging. Short-term aging, according to Polish Technical Requirements WT-2 [39], included the following stages: seasoning the mixture at 135 °C for two hours, raising the temperature to 140 °C and maintaining it for an hour before compacting the samples in a gyratory compactor (IPC Global, Boronia, Australia). This procedure is intended to simulate changes in bitumen during the production, laying and compaction of the asphalt mixture on the construction site. Long-term aging was performed according to the following procedure: after short-term aging, the mixture was placed in containers in a dryer with airflow at a temperature of 85 °C for a period of 120 h. This method is consistent with the recommendations of the literature [40,41], where loose mixture aging was recommended as more reliable than that of compacted samples. As was stated in [42], a temperature during aging higher than 95 °C may have an adverse effect on the bitumen oxidation process. The conditioning procedure used in the tests corresponds to the aging that occurs in the pavement over several to a dozen years of operation, depending on the climatic conditions and the depth of the layer location.

After the aging procedure, the mixtures were placed in a mixer, heated to 140 °C, placed in 150 mm diameter molds and then compacted by gyration to the target height of the samples of 150 mm. The final content of the air voids in the compacted samples was set at 4%, which avoids the influence of the physical characteristics of the samples on the tested parameters used to assess fracture toughness. The designations of the individual test series of asphalt mixture are given in Table 5.

From each mixture designation, five cylindrical samples were compacted according to PN-EN 12697-31 [43]. Then, from each sample with a height of 150 mm, two cylinders with a height of 50 mm were cut out using a double-disk saw. The resulting cylinders were cut along their diameter. In the last step, the samples were cut along the symmetry axis of each half, obtaining three series of 6 samples each, differing in the depth of the cut, and leaving two uncut samples as a reserve for testing. In this way, twenty samples were prepared for SCB testing for each of the four designated groups (mixture type and aging conditions).

**Figure 3:**
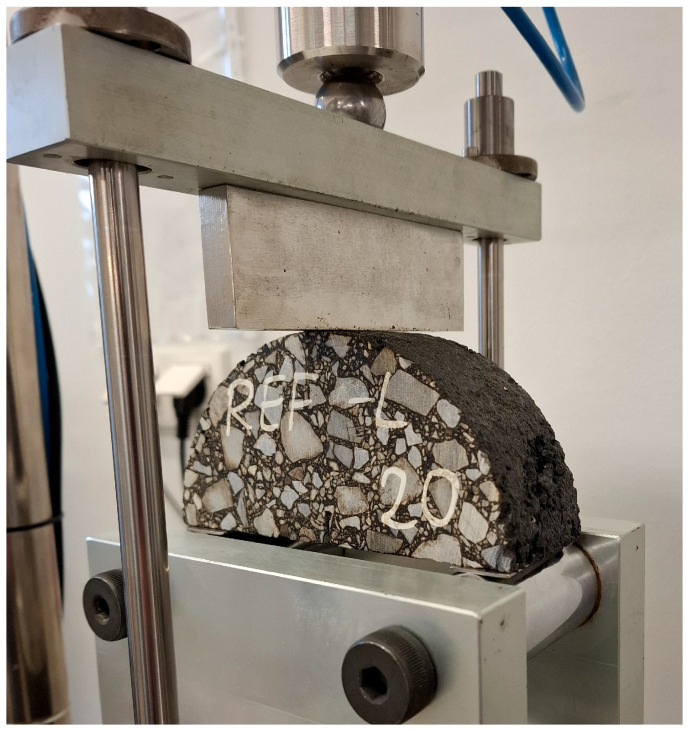
SCB test setup.

**Figure 4:**
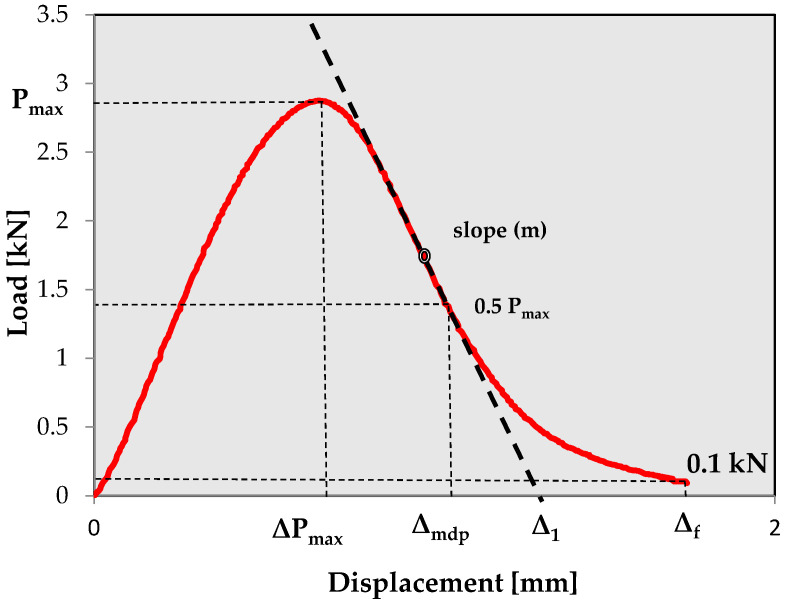
Example of load–displacement scheme after SCB test.

SCB samples were kept at a temperature of 20 °C in the air chamber for a minimum of two hours and then were tested using the SCB jig in the Marshall press at a load rate of 1.25 mm/min, at a temperature of 20 °C. The test conditions align with those recommended by Nsemgiyumva, Kim and You [34]. The measurement continued until the force dropped to 0.1 kN. Figure 3 shows the SCB test setup, while Figure 4 presents the balance path diagram (load–displacement) along with the selection of sample parameters derived from this chart.

**Figure 5:**
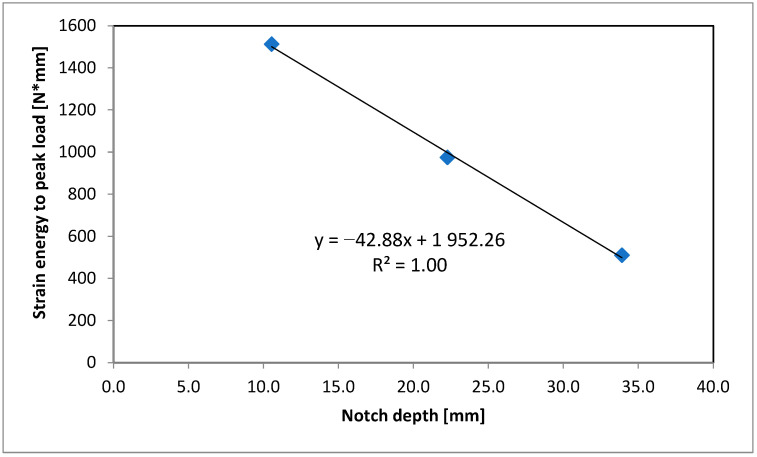
Results for slope from plot of strain energy vs. notch depth for RAS_S mixture.

To determine the critical strain energy release rate J-integral (Jc), samples with three notch depths were tested (10 mm, 22 mm and 34 mm). An example of a designation of the slope of the linear regression from the plot of strain energy vs. the notch depth used for Jc calculation for the RAS_S mixture is presented in Figure 5 (each point represents average value of six samples for specific notch depth).

The SCB test results were used to determine the parameters used to assess the crack resistance of the asphalt mixtures according to Formulas (1) to (12).

(1) $$\sigma_{max}=\frac{P_{max}}{D*t}$$

where the following hold: σ_max_—maximum bending stress, P_max_—peak load value, D—sample diameter, t—sample thickness.

(2) $$\varepsilon_{max}=\frac{\Delta_{Pmax}}{h}*100$$

where the following hold: ε_max_—strain at peak load in %, Δ_Pmax_—vertical displacement at peak load in mm, h—sample height in mm.

(3) $$SM=\frac{P_{max}}{\Delta_{Pmax}}$$

where Sm is the secant modulus.

(4) $$K_{IC}=\sigma_{max}*Y_{1}*\sqrt{\pi *a}$$

(5) $$Y_{1}=4.782-1.219*\frac{a}{r}+0.063*exp\;(7.045*\frac{a}{r})$$

where the following hold: K_IC_—fracture toughness (N/mm^1.5^), Y_1_—normalized mode I stress intensity factor, a—notch depth, r—specimen radius of height.

(6) $$J_{C}=-\frac{1}{t}*\frac{dU}{da}$$

where the following hold: J_C_—the critical value of the J-integral, U—strain energy to peak load according to Equation (7), dU/da—change in strain energy with change in notch depth.

(7) $$U=\sum_{i=1}^{n}\left(x_{i+1}-x_{i})*y_{i}+0.5*(x_{i+1}-x_{i})*{(y_{i+1}-y}_{i}\right)$$

where x_i_ is the displacement recorded, y_i_ is the load recorded and n is the point at which the load achieved maximum value.

(8) $$G_{f}=\frac{W_{f}}{t*(h-a)}$$

where the following hold: G_f_—fracture energy, W_f_—total strain energy (calculated according to Equation (7); n is the point at which the load decreases to 0.1 kN).

(9) $$FI=\frac{G_{f}}{\left|m\right|}*A$$

where the following hold: FI—flexibility index, m—the value of the slope at the inflection point after peak load, A—calibration factor coefficient, where the default is 0.01.

(10) $$CRI=\frac{G_{f}}{P_{max}}$$

where CRI is the cracking resistance index.

(11) $$TI=G_{f,\;post\;peak}*(\Delta_{mdp}-\Delta_{Pmax})*10^{-3}$$

where the following hold: TI—toughness index, G_f_; post-peak—post-peak fracture energy according to Equation (12); Δ_mdp_—displacement at 50% of the peak load.

(12) $$G_{f,\;post\;peak}=\frac{W_{f}-U}{t*(h-a)}$$

## 3. Results

### 3.1. Laboratory Results

**Figure 6:**
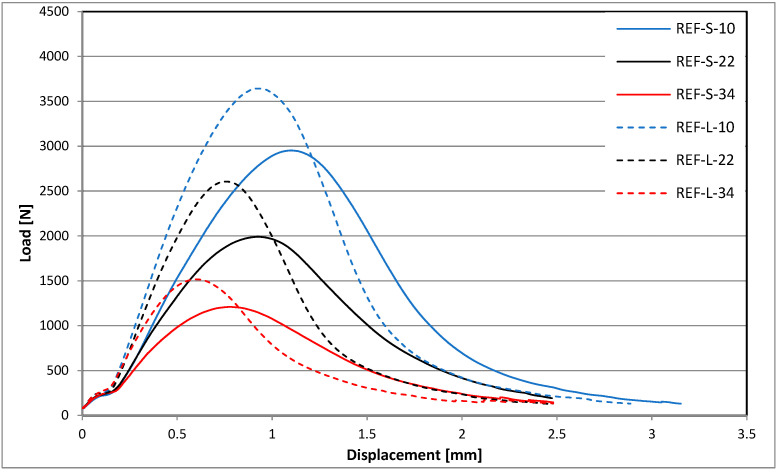
Force-displacement charts for REF mixtures.

**Figure 7:**
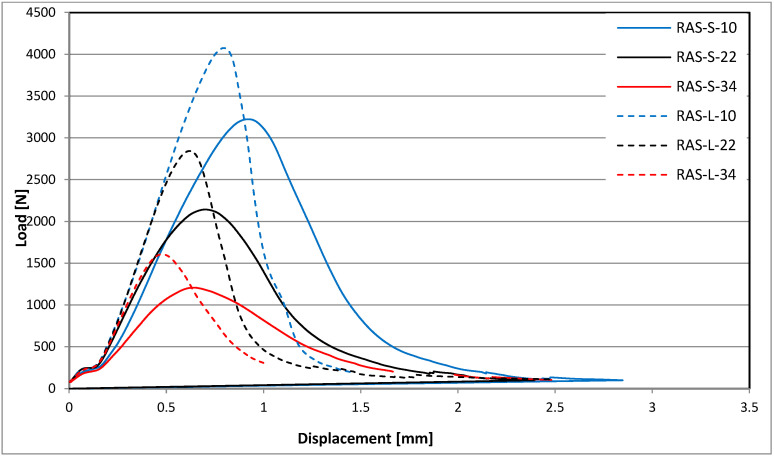
Force-displacement charts for RAS mixtures.

The average shapes of the force-displacement charts for the individual tested mixtures are different, as shown in Figure 6 (REF) and Figure 7 (RAS).

The results of selected parameters determined from the SCB test are given in Table 6.

For the determined cracking parameters of individual mixtures, analyses of the significance of differences were performed using the Statgraphics Plus v. 5.1 computer program [44], which allowed us to assess whether the obtained results differ significantly. An Anova function of multiple range tests was used, applying the least square differences option and using an assumed confidence level of 0.95. After analyzing the parameters given in Table 6, the largest differences were found for the secant modulus (SM), presented in Table 7.

**Figure 8:**
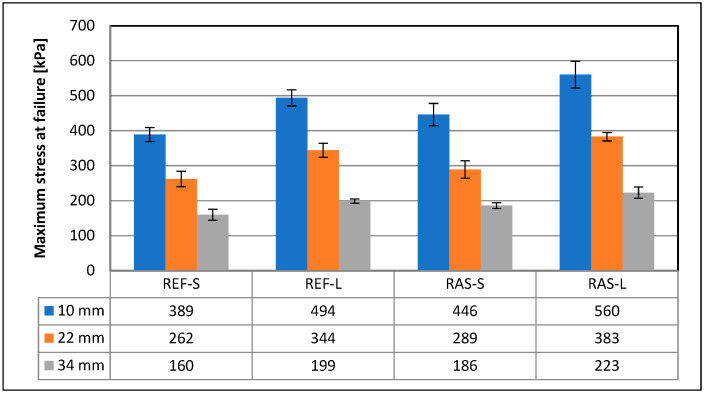
Maximum bending stress—σ_max_.

**Figure 9:**
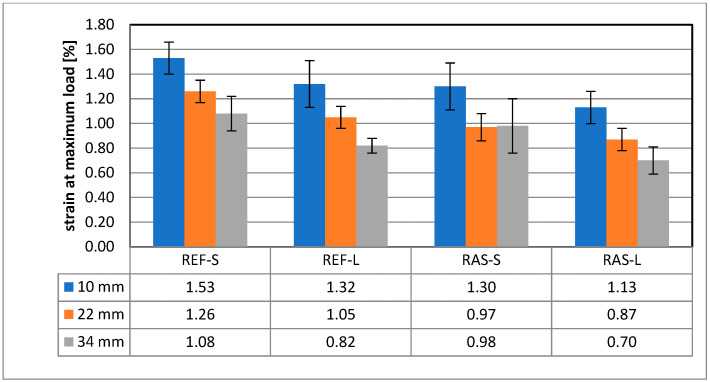
Strain at maximum load—ε_max_.

**Figure 10:**
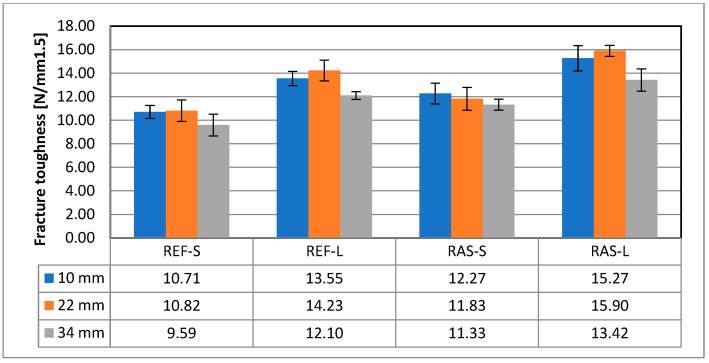
Fracture toughness—K_IC_.

Other parameters of the mixtures designated according to PN-EN 12697-44 [45] are shown in Figure 8, Figure 9 and Figure 10. Statistical test results for the K_IC_ parameter are shown in Table 8.

**Figure 11:**
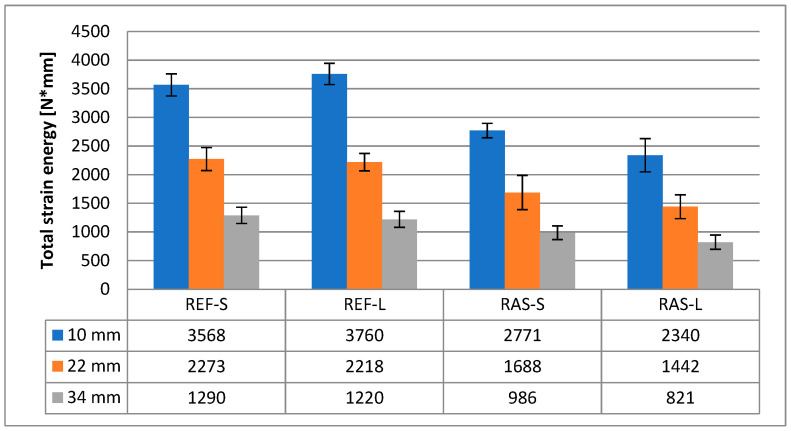
Total strain energy—W_f_.

**Figure 12:**
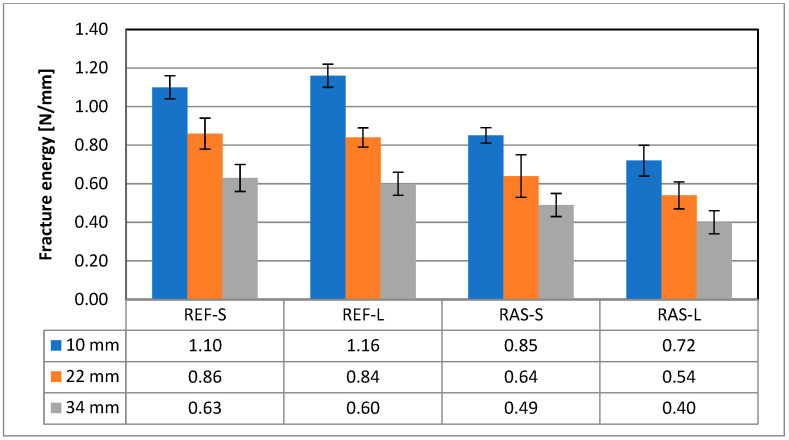
Fracture energy—G_f_.

For the assessment of energy parameters, total strain energy (W_f_) and fracture energy (G_f_) were calculated. As shown in Figure 11 and Figure 12, for mixtures with added RASs, the parameters for assessing crack resistance are worse than for the reference mixture. The results of statistical analyzes of both above-mentioned parameters were similar; for example, in Table 9, they are presented for the total strain energy (W_f_).

**Figure 13:**
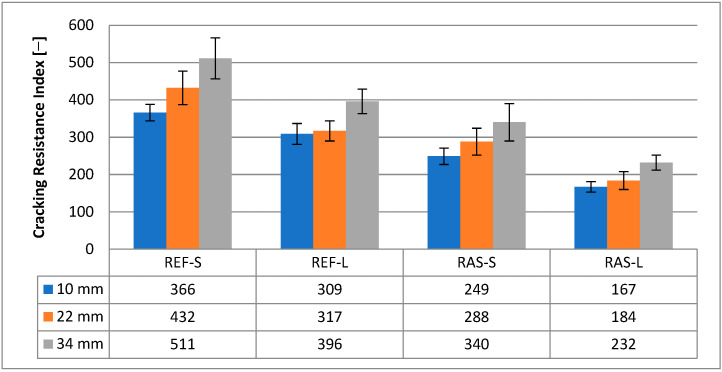
Cracking resistance index—CRI.

The cracking resistance index (CRI), which corresponds to the value of the fracture energy divided by the maximum force, is presented in Figure 13. The statistical analyses presented in Table 10 indicate the importance of differentiating the tested mixtures using the CRI.

**Figure 14:**
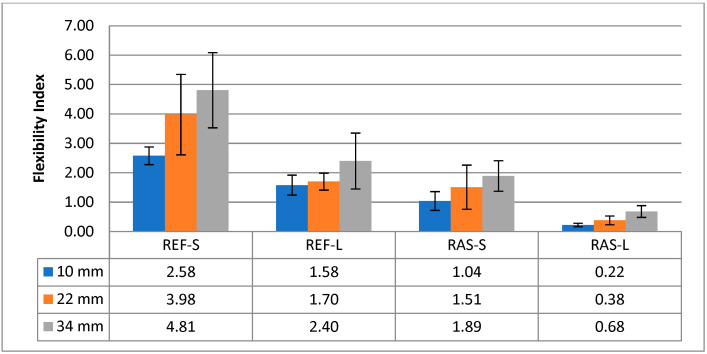
Flexibility index—FI.

**Figure 15:**
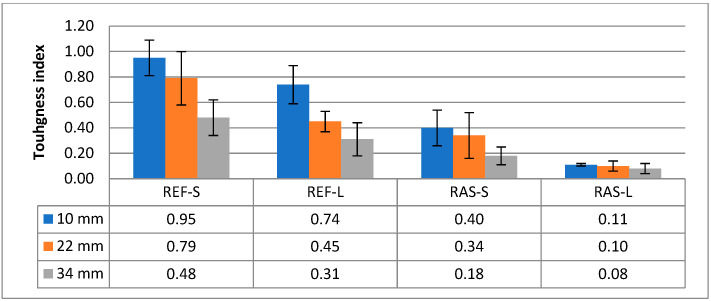
Toughness index—TI.

The list of parameters describing the post-peak load, such as FI and TI, is shown in Figure 14 and Figure 15, while their statistical analyses are presented in Table 11 and Table 12. These parameters allow for the differentiation of the mixtures in terms of crack resistance, both in terms of the addition of RASs and the aging conditions of HMA.

### 3.2. Numerical Results

In order to provide a deeper insight into the specimen performance, we also carried out a digital image correlation (DIC) analysis together with a numerical analysis based on the image processing. Laboratory results presented in the previous section provide a macroscopic response of the specimen. Taking into account the internal microstructure of the asphalt mixture allows for a more detailed analysis. In this study, we present the results of a twofold approach. Firstly, we show the DIC results obtained during the SCB test. For the sake of brevity, only a set of selected results is presented, to illustrate the potential benefits of this approach. Secondly, we also present the application of image processing for the asphalt mixture specimens. This technique can be profitable in the context of further numerical modeling. Selected results of this approach are also presented.

#### 3.2.1. Digital Image Correlation

Optical measurements were conducted using a Nikon D5300 digital single-lens reflex (DSLR) camera (Nikon, Tokyo, Japan), equipped with a Sigma 17–50 mm f/2.8 EX DC OS HSM and Tamron AF 70–300 mm f/4–5.6 DI LD lenses, chosen for its minimal radial distortion to ensure high-quality images for subsequent analysis. The sample surface was photographed at consistent intervals using an intervalometer Newell MC-DC2 (Newell, Łódź, Poland). Each image had a resolution of 6000 pixels horizontally and 4000 pixels vertically, resulting in a 24 Mpx image size. The camera was mounted on a tripod with a micrometer head and positioned perpendicularly to the sample surface. The focal length was 28 mm for the Sigma lens when imaging the entire sample surface and approximately 200 mm for the Tamron lens when the images focused on the area around the notch, where strain concentration occurred and the failure of the sample was initiated. Also, an ISO value between 100 and 200 and a shutter speed ranging from 1/50 to 1/25 s were set. To maintain a high shutter speed and avoid color distortions, a strong LED light source with a color temperature range of 4000–5700 K was employed. To enable the determination of structural displacements from subsets of a digital image of the sample surface, the Digital Image Correlation (DIC) method was employed. This method was implemented in the proprietary CivEng Vision software developed at CUT for optical measurements and was used to process all the captured photos—see Tekieli et al. [46] for details. The core principle of using cross-correlation to measure shifts in datasets involves comparing fragments of the digital image with its surroundings to identify the displacement of the analyzed fragment within the Cartesian coordinate system. The pixel displacement can then be easily converted into real metric values by analyzing the lens parameters, its distance from the sample surface, or by scaling based on the known size of an object in the calibration image. By measuring the displacement of two independent subsets of the image, the engineering strain values can be determined for a specific area. Additionally, by overlaying a grid of markers (subsets) on the sample surface in the image, it is possible to create a displacement map (field) and, subsequently, a deformation field. In this study, zero-mean normalized cross-correlation (ZNCC) was used for marker tracking. To achieve measurement resolution comparable to traditional sensors (such as strain gauges), subpixel measurement was employed, which was obtained by interpolating pixel values to intermediate values assigned to subpixels.

It is also important to remember the limitations of optical methods and the potential measurement errors associated with them. These errors may result from improper image registration during testing. A key factor is the proper illumination of the testing sample with an appropriate light source, which is why neutral-colored LED lighting was used. Strong illumination of the sample minimizes the aperture opening time, ensuring that any movement of the sample during photography does not affect image imperfections. Additionally, it allows for setting a very low ISO of 200 or even 100, which reduces digital image noise. To minimize errors caused by image distortion, a high-quality lens with minimal radial distortions was used, and the camera-to-sample distance was adjusted to allow for a focal length setting that eliminates barrel and pincushion distortions. The focal length was set at the transition point between the barrel and pincushion distortions, where the image is closest to the ideal and almost free of distortions.

**Figure 16:**
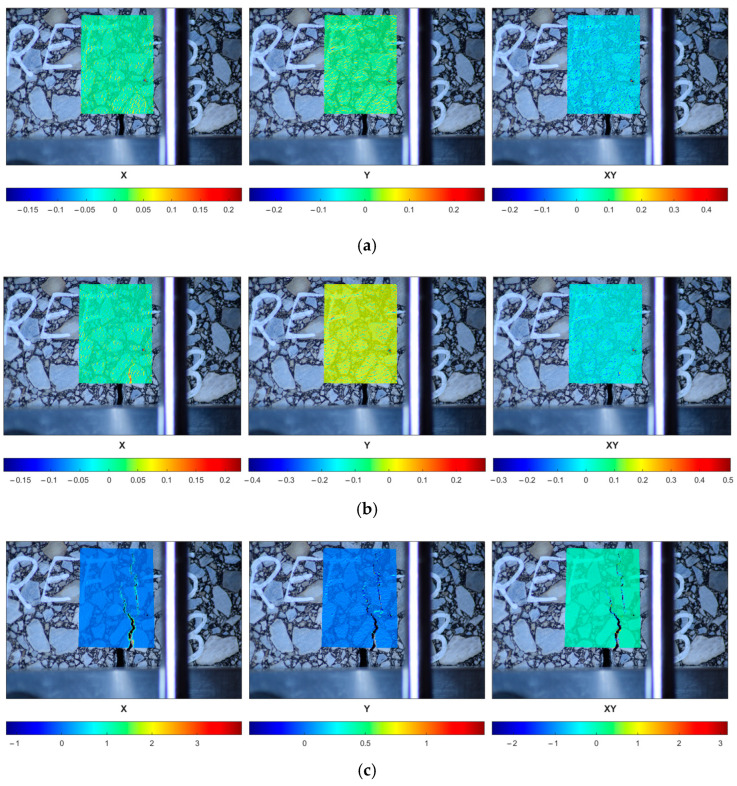
REF-S-10 specimen performance in peak load range—strain components (ε_xx_, ε_yy_, ε_xy_ shown in columns, consecutively) plotted for selected time instances: (**a**) t = 54 s, (**b**) t = 58 s, (**c**) t = 90 s.

**Figure 17:**
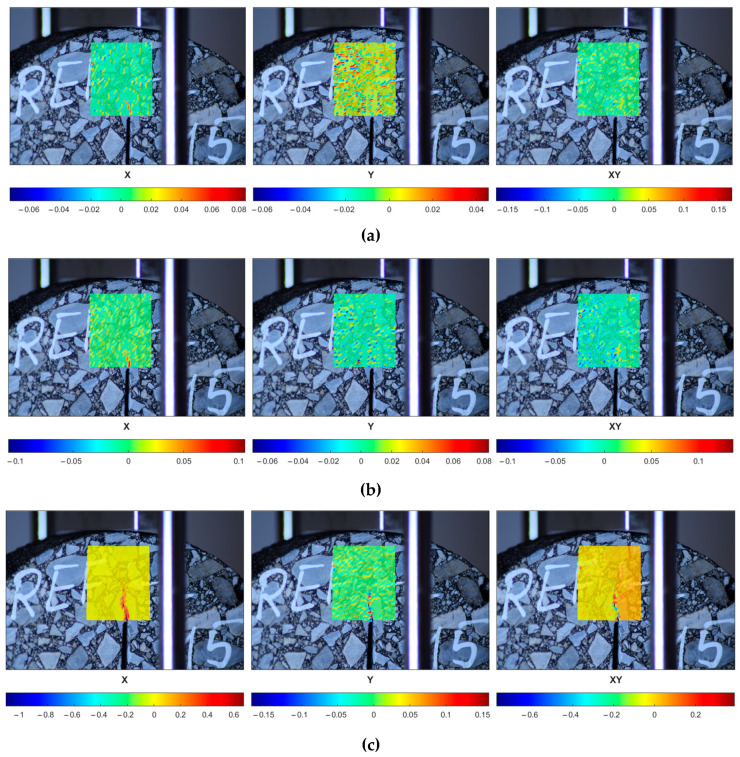
REF-L-10 specimen performance in peak load range—strain components (ε_xx_, ε_yy_, ε_xy_ shown in columns, consecutively) plotted for selected time instances: (**a**) t = 48 s, (**b**) t = 52 s, (**c**) t = 80 s.

**Figure 18:**
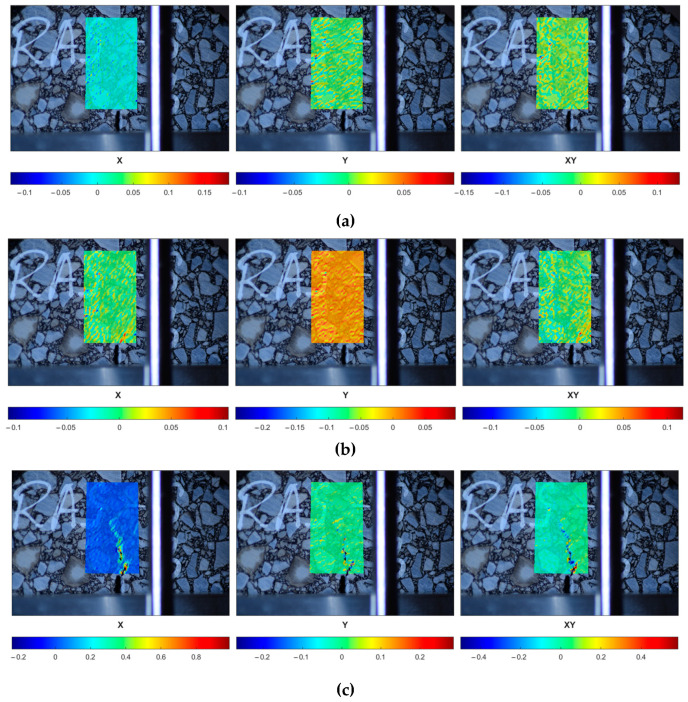
RAS-S-10 specimen performance in peak load range—strain components (ε_xx_, ε_yy_, ε_xy_ shown in columns, consecutively) plotted for selected time instances: (**a**) t = 50 s, (**b**) t = 70 s, (**c**) t = 100 s.

**Figure 19:**
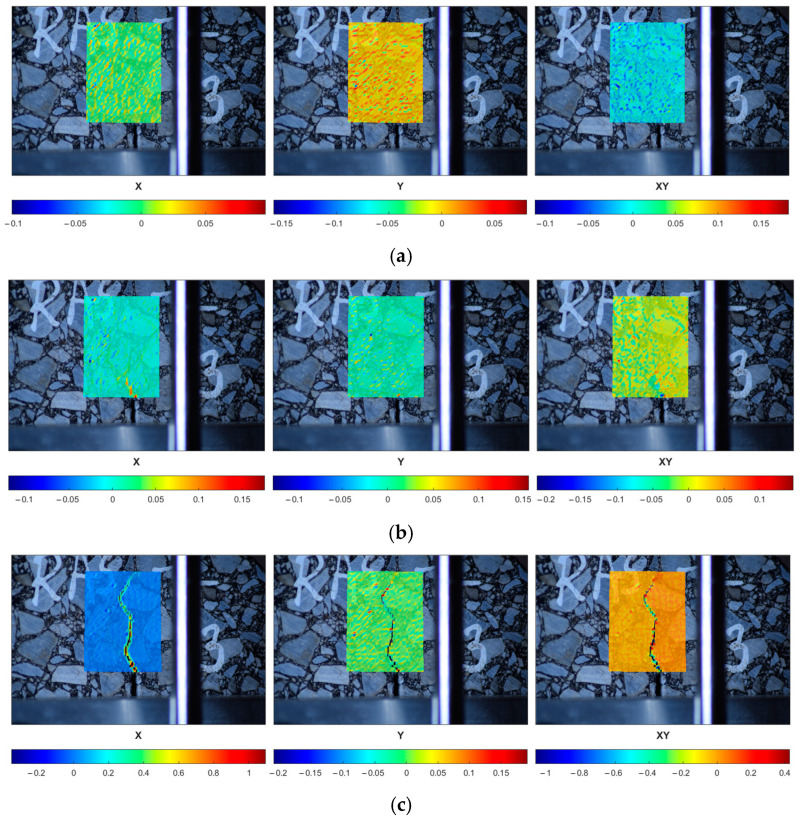
RAS-L-10 specimen performance in peak load range—strain components (ε_xx_, ε_yy_, ε_xy_ shown in columns, consecutively) plotted for selected time instances: (**a**) t = 52 s, (**b**) t = 58 s, (**c**) t = 66 s.

In Figure 16, one can observe the performance of the selected reference specimen after the short-term aging (REF-S) in a peak load range. The initial strain localization (the first row) is followed by the crack initiation (the second row) and its further propagation (the third row). In Figure 17, the response of a sample subjected to long-term aging (REF-L) is shown. Figure 18 and Figure 19 present the response of the RAS specimens subjected to short- and long-term aging (RAS-S and RAS-L, respectively). One can observe different behavior for each specimen type, which corresponds with Figure 6 and Figure 7. Specifically, the peak load occurs at different time instances. This is specified in the figures and can be seen in the captions of Figure 16, Figure 17, Figure 18 and Figure 19.

DIC analysis allows for crack path tracking from the crack initiation to the specimen failure. Since the DIC provides the quantitative analysis, the crack initiation time can be reported.

#### 3.2.2. Image Processing

**Figure 20:**
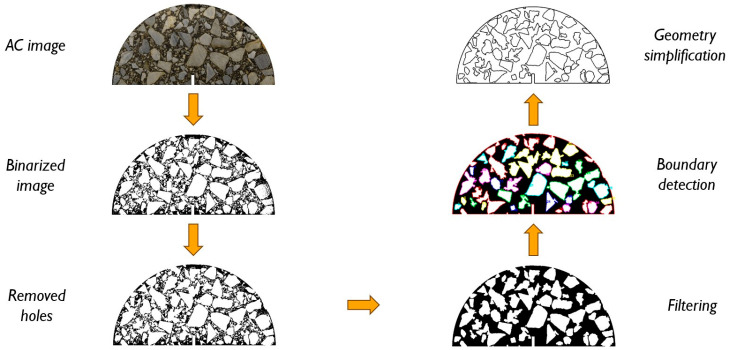
A flowchart of the image processing for the SCB test.

As a starting point for our future research program, we also present the initial results of the finite element analysis of the arbitrarily selected specimen in a plane strain state. The key part of this approach is the image processing technique that allows for microstructure recognition and the modeling of the domain, accounting for its heterogeneity. Its flowchart is schematically presented in Figure 20. The method has been developed, e.g., by Klimczak, Jaworska and Tekieli [47], with a particular focus on microstructure geometry simplification for the facilitation of further finite element analysis.

**Figure 21:**
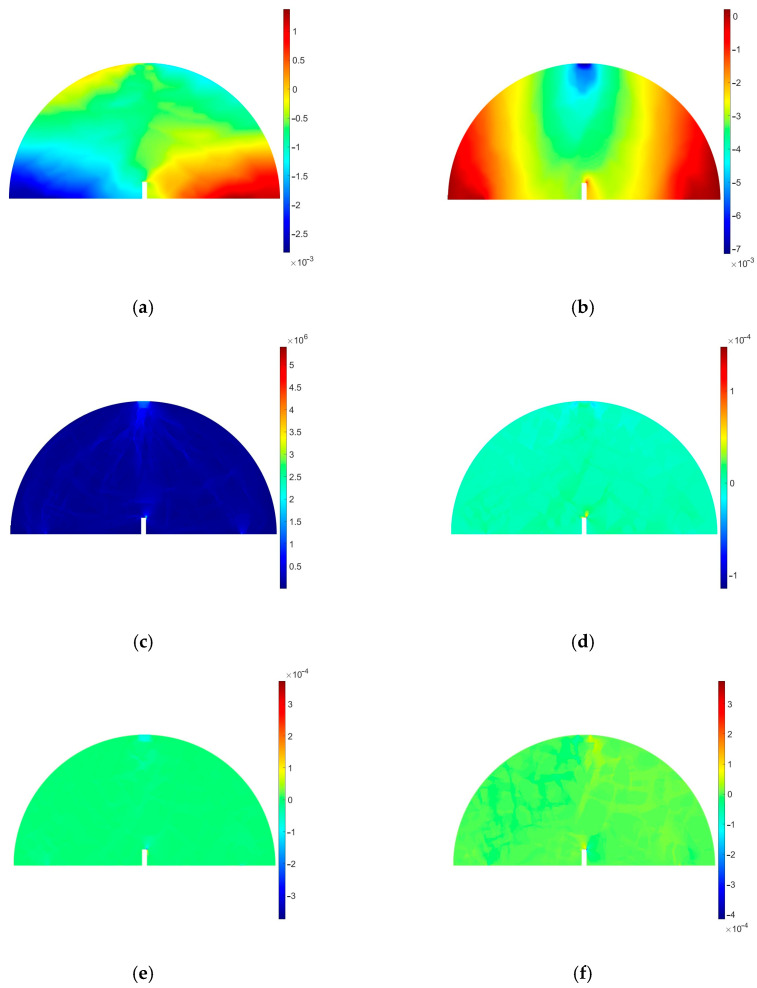
Numerical results of the finite element analysis: (**a**) displacement component u_x_ [m], (**b**) displacement component u_y_ [m], (**c**) von Mises stress [Pa], (**d**) strain tensor component ε_xx_ [−], (**e**) strain tensor component ε_yy_ [−], (**f**) strain tensor component ε_xy_ [−].

Starting with a high-quality image, we convert it to grayscale and perform its binarization. After the removal of “holes” from the inclusions (aggregate particles), filtering is applied to exclude from the analysis inclusions smaller than a threshold value. When the inclusion boundaries are detected, they can possibly be subjected to further geometry simplification. This is due to the reduction in the finite element mesh density. In Figure 21, numerical results for a selected specimen are presented. They refer to the pre-peak behavior since the linear elastic material model was assumed. Material parameters for the inclusion were assumed for the data found in the literature for dolomite, whereas the binder parameters were obtained using the curve-fitting approach. Since the linear elastic model was assumed, no crack could be analyzed at this step. A further enhancement of the approach is to use cohesive zone elements to model the possible cracking process. Also, a viscoelastic material model should be applied for the bitumen phase.

Material data for the linear elastic analysis (pre-peak behavior) are presented in Table 13. In Figure 3, the SCB test setup is shown. In the numerical analysis, boundary conditions were to be reconstructed by fixing vertical displacements along the supports (u_y_ displacement component is set to 0) and applying the vertical compressive load along a part of the top edge. The microstructure of the analyzed sample was reconstructed based on the high-quality image shown in Figure 20. Therein, the consecutive image processing step results are also presented. Triangular finite elements with a linear approximation were used in the analysis.

In Figure 21, the numerical results are presented for the specimen, accounting for the microstructure heterogeneity. As stated above, only linear elastic behavior is studied in this test. These specific results presented in Figure 21 refer to a time t = 3 s. However, due to the adopted model, they can only be considered as initial results. A nonlinear material model for the bitumen and contact between the microstructure constituents would facilitate a complete numerical analysis.

Since the linear elastic model was assumed for both the aggregate and the binder phase, no cracking is modeled. However, one can observe the strain localization close to the notch corners. It coincides with the crack initiation area observed in Figure 16, Figure 17, Figure 18 and Figure 19. Our future research effort will be to apply the viscoelastic material model for the bitumen and also to use the cohesive zone model (CZM) to model the adhesion between the aggregate and bitumen. Such a study can provide a more detailed description of the asphalt mixture response.

## 4. Discussion

The reference mixture subjected to short-term aging (REF-S) is characterized by the lowest maximum forces with the highest values of displacement parameters (such as Δ_Pmax_, ε_max_, Δ_mdp_), while the mixtures with added RASs after long-term aging (RAS-L) are characterized by the highest values of transmitted force with the smallest displacements. The behavior of the reference mixture after long-term aging (REF-L) is within the above-mentioned range parameters, similar to those of mixtures with added RASs after short-term aging (RAS-S).

RAS samples, compared to the reference mixture samples, achieved an increase in the secant modulus parameter by approx. 32 ÷ 44%, and its increase was also found with the extension of the aging time (by approx. 45 ÷ 65%), with the highest value of the SM parameter obtained for the samples with the smallest tested notch depth, which was 10 mm.

The use of K_IC_ and J_C_ parameters to assess the crack resistance of mixtures is only effective in distinguishing reference mixtures from those with added RASs, and the values of these parameters obtained after long-term aging were higher than those obtained for samples after short-term aging, which is inconsistent with the expected trend. This indicates the lack of usefulness of using these parameters to assess the crack resistance of asphalt concrete subjected to various aging conditions.

Correct differentiation between the tested mixtures was achieved using energy parameters, i.e., total strain energy (W_f_) and fracture energy (G_f_). These parameters differentiate the results between RAS mixtures and the reference mixture well, but they do not indicate statistically significant differences between samples from the same mixtures subjected to different aging conditions.

The parameters based on post-peak slope analysis, such as FI and TI, turned out to be very sensitive to the type of mixture tested (composition, aging conditions). The decrease in the FI parameter after long-term aging was approximately twofold for the reference mixture and 3–5 times for the mixture with added RASs. Considering samples after long-term aging, the toughness index decreased by 2.2–3.6 times for the RAS mixture and about 1.3–1.8 times for the REF mixture. In the case of the CRI parameter, the scope of changes was smaller, although the direction was consistent with the FI and TI parameters.

Ultimately, among the use of SCB test parameters to assess the cracking resistance of mixtures, the most conclusive is the use of parameters based on the post-peak load, which was confirmed in the TRB Report [25] and the work conducted by Haslett [33]. At the same time, the TI and the FI parameters are characterized by a relatively large dispersion of results, which makes it difficult to determine the significance of the differences. For the FI parameter, the COV values are on average 25% (for REF mixtures) and 34% (for RAS mixtures), while for the TI parameter, the COV values are 25% (for REF mixtures) and 38% (for RAS mixtures). Regarding FI and TI parameters after short-aging for mixtures with RAS, a decrease of approximately 60% was achieved compared to the reference mixture. The FI parameter after long-term aging exhibits a decrease equal to approximately 50% for REF mixtures and approximately 70% for RAS mixtures compared to its short-term aging values.

Based on the statistical analyses, it was found that the most effective parameter in differentiating the tested series of samples was the CRI parameter, for which relatively low variability rates of approximately 10% (in the range of 6.0—14.7%) were found. Mixtures with added RASs show a decrease in the CRI parameter compared to the reference mixture, depending on the aging conditions: by approximately 33% for short-term aging and approximately 43% for long-term aging.

## 5. Conclusions and Recommendations

Based on the research and analyses carried out, the following conclusions and recommendations can be formulated:

- In this study, the application of a civil engineering waste product (RASs) was analyzed, which is in line with current trends based on sustainability.
- RAS mixtures subjected to long-term aging stiffened significantly and, therefore, became much more susceptible to cracking compared to the reference one. To solve the above-mentioned problem, optimization of the type and amount of rejuvenator is planned in the future.
- SCB test parameters determined in accordance with the European standards [45], i.e., K_IC_ and J_C_, do not allow for a proper assessment of AC mixtures.
- Correct differentiation between the mixture composition was achieved using energy parameters, i.e., total strain energy (W_f_) and fracture energy (G_f_), but in the case of aging conditions, evaluation based on them is not appropriate, which is consistent with the conclusions of the work conducted by Jiang et al. [49].
- The post-peak load indexes, i.e., FI and TI, turned out to be the most sensitive to the aging conditions of the mixtures; however, due to the relatively large dispersion of the results (COV is about 30%), they may be less conclusive in terms of assessing the statistical significance of differences between the tested series, especially for a small series of samples.
- The best sensitivity in terms of differentiating the tested mixtures was observed for the CRI parameter, the advantage of which is the relatively small scatter of the obtained results (COV is about 10%).
- The image processing techniques presented in the paper can be used for the enhancement of laboratory experiments due to the possibility of a more detailed description of analyzed phenomena. Further research efforts will reconstruct a fully tridimensional specimen microstructure and apply cohesive zone elements to model the fracture phenomena numerically.

It is also worth noting that the use of optical measurements allowed for an expansion of the spectrum of obtained results and better tracking of the phenomena occurring in the tested samples during tests. In this case, optical measurements would not fully replace traditional measurements, but they allowed for an extension of their effectiveness and provided a better description of the research.

## Figures and Tables

**Table 1:** Composition of RAS and extracted bitumen properties.

Sieve Size [mm]	Grading Curve	Bitumen Properties
5.6	100	
4.0	83.8	
2.0	72.0	
0.5	56.4	
0.125	46.8	
0.063	40.8	
Bitumen amount [%]		34.1
Penetration [0.1 mm]		17
Softening Point [°C]		102.8

**Table 2:** Components of asphalt concrete mixtures.

O.N.	Components	Participation in AC [%]	
		Reference	RAS
1	Limestone filler	4.3	3.34
2	Sand 0/2	9.56	9.54
3	Dolomite 0/4	26.77	24.99
4	Dolomite 4/8	15.77	15.74
5	Dolomite 8/16	39.2	39.1
6	RAS	-	4.0
7	Paving bitumen 50/70	4.4	3.3

**Table 3:** Composition of AC samples.

Sieve Size [mm]	Grading Curve	
	REF	RAS
22.4	100	100
16.0	98.2	98.2
11.2	78.4	78.4
8.0	58.2	58.2
5.6	47.5	47.6
4.0	42.7	42.3
2.0	31.9	31.8
0.5	14.2	14.5
0.125	7.3	7.5
0.063	6.3	6.4
Bitumen recovered [%]	4.41	4.56

**Table 4:** Test results of fresh bitumen as well as binder recovered from mixtures after aging.

Bitumen Results	Fresh Bitumen 50/70	REF-S	REF-L	RAS-S	RAS-L
Penetration [0.1 mm]	61	28	23	24	16
R&B [°C]	49.2	62.2	65.9	66.9	80.8

**Table 5:** Asphalt mixture designations.

Mixture Designation	Reclaimed Asphalt Additive	Aging Conditions
REF_S	(-)	Short-term (S)
RAS_S	(RAS)	Short-term (S)
REF_L	(-)	Long-term (L)
RAS_L	(RAS)	Long-term (L)

**Table 6:** SCB parameters (mean values).

Mixture Type	Notch Depth [mm]	Pmax [kN]	ΔPmax [mm]	Δmdp [mm]	SM [N/mm]	U [N*mm]	dU/da [N]	J_C_ [N/mm]
REF_S	10	3.00	1.13	1.65	2.68	1760	−56.29	1.10
	22	2.00	0.93	1.53	2.18	974		
	34	1.23	0.80	1.40	1.58	510		
RAS_S	10	3.41	0.96	1.27	3.60	1512	−42.88	0.83
	22	2.22	0.71	1.07	3.15	783		
	34	1.43	0.72	1.04	2.07	414		
REF_L	10	3.78	0.98	1.37	3.95	1924	−61.20	1.19
	22	2.65	0.77	1.15	3.45	1023		
	34	1.53	0.60	1.02	2.53	474		
RAS_L	10	4.30	0.83	0.97	5.23	1597	−52.42	1.00
	22	2.96	0.64	0.80	4.64	835		
	34	1.72	0.52	0.69	3.41	363		

**Table 7:** Multiple range test results for SM parameter (difference between groups).

Compared Mixtures	Difference for Notch Depth		
	10 mm	22 mm	34 mm
REF_S—RAS_S	−0.92 *	−0.97 *	−0.49
REF_S—REF_L	−1.27 *	−1.27 *	−0.96 *
REF_S—RAS_L	−2.55 *	−2.46 *	−1.83 *
RAS_S—REF_L	−0.35	−0.29	−0.47
RAS_S—RAS_L	−1.63 *	−1.48 *	−1.34 *
REF_L—RAS_L	−1.28 *	−1.18 *	−0.88 *
Statistically significant limit	0.64	0.42	0.55

* denotes a statistically significant difference for a calculated limit.

**Table 8:** Multiple range test results for K_IC_ parameter (difference between groups).

Compared Mixtures	Difference for Notch Depth		
	10 mm	22 mm	34 mm
REF_S—RAS_S	1.56 *	−1.01 *	−1.75 *
REF_S—REF_L	−1.28 *	−3.40 *	−2.52 *
REF_S—RAS_L	−3.00 *	−5.07 *	−3.84 *
RAS_S—REF_L	−2.84 *	−2.40 *	−0.77
RAS_S—RAS_L	−4.56 *	−4.07 *	−2.09 *
REF_L—RAS_L	−1.73 *	−1.67 *	−1.32 *
Statistically significant limit	0.97	1.00	0.88

* denotes a statistically significant difference for a calculated limit.

**Table 9:** Multiple range test results for Wf parameter (difference between groups).

Compared Mixtures	Difference for Notch Depth		
	10 mm	22 mm	34 mm
REF_S—RAS_S	−798 *	585 *	304 *
REF_S—REF_L	−192	55	70
REF_S—RAS_L	431 *	831 *	469 *
RAS_S—REF_L	−989 *	−531 *	−234 *
RAS_S—RAS_L	431 *	246	165
REF_L—RAS_L	1420 *	776 *	399 *
Statistically significant limit	364	266	167

* denotes a statistically significant difference for a calculated limit.

**Table 10:** Multiple range test results for CRI parameter (difference between groups).

Compared Mixtures	Difference for Notch Depth		
	10 mm	22 mm	34 mm
REF_S—RAS_S	−116 *	144 *	172 *
REF_S—REF_L	−59 *	114 *	116 *
REF_S—RAS_L	−82 *	248 *	280 *
RAS_S—REF_L	57 *	−29	−56 *
RAS_S—RAS_L	199 *	104 *	108 *
REF_L—RAS_L	142 *	133 *	164 *
Statistically significant limit	32	41	52

* denotes a statistically significant difference for a calculated limit.

**Table 11:** Multiple range test results for FI parameter (difference between groups).

Compared Mixtures	Difference for Notch Depth		
	10 mm	22 mm	34 mm
REF_S—RAS_S	1.54 *	2.47 *	2.92 *
REF_S—REF_L	1.00 *	2.29 *	2.41 *
REF_S—RAS_L	2.36 *	3.61 *	4.13 *
RAS_S—REF_L	−0.54 *	−0.19	−0.51
RAS_S—RAS_L	0.82 *	1.13 *	1.20 *
REF_L—RAS_L	1.36 *	1.32 *	1.72 *
Statistically significant limit	0.33	0.96	1.08

* denotes a statistically significant difference for a calculated limit.

**Table 12:** Multiple range test results for TI parameter (difference between groups).

Compared Mixtures	Difference for Notch Depth		
	10 mm	22 mm	34 mm
REF_S—RAS_S	0.55 *	0.45 *	0.30 *
REF_S—REF_L	0.21 *	0.35 *	0.17 *
REF_S—RAS_L	0.84 *	0.69 *	0.40 *
RAS_S—REF_L	0.21 *	−0.10	0.12
RAS_S—RAS_L	0.29 *	0.24 *	0.10
REF_L—RAS_L	0.63 *	0.35 *	0.23 *
Statistically significant limit	0.14	0.18	0.13

* denotes a statistically significant difference for a calculated limit.

**Table 13:** Material parameters for the linear elastic finite element analysis [48].

Parameter	Unit	Value
Dolomite Young modulus	GPa	38.16
Dolomite Poisson ratio	-	0.24
Binder Young modulus	GPa	0.89
Binder Poisson ratio	-	0.25

## Data Availability

The original contributions presented in this study are included in the article. Further inquiries can be directed to the corresponding author.

## Abbreviations

List of abbreviations in alphabetic order

Abbreviation	Meaning
AC	Asphalt concrete
BCI	Balanced cracking index
COV	Coefficient of variation
CRI	Cracking resistance index
CUT	Cracow University of Technology
CZM	Cohesive zone model
DIC	Digital image correlation
FEA	Finite element analysis
FEM	Finite element method
FI	Flexibility index
MWAS	Manufacture waste asphalt shingles
RASs	Reclaimed asphalt shingles
REF	Reference mixture
SCB	Semi-circular bending
SM	Secant modulus
TOASs	Tear-off asphalt shingles
TI	Toughness index
ZNCC	Zero-mean normalized cross-correlation
